# Supplementary material for: Non-canonical IL‑22 receptor signaling remodels the oral mucosal barrier during Candida albicans immunosurveillance
Source: Nat Commun. 2026 Apr 3;17:4823. doi: 10.1038/s41467-026-71459-z (PMC13223278; doi:10.1038/s41467-026-71459-z)
Supplement: Supplementary file 1 — Supplementary Information [file 41467_2026_71459_MOESM1_ESM.pdf]

Non-canonical IL-22 receptor signaling remodels the oral mucosal barrier during *Candida albicans* immunosurveillance

Nicolas Millet[1,2,†], Jinendiran Sekar[1,2], Norma V. Solis[1,2], Jian Miao[1,2], Antoine Millet[2,3], Felix E.Y. Aggor[4], Asia Wildeman[1,2], Melissa E. Cook[4], Amirhossein Davari[5], Brian M. Peters[6], Michail S. Lionakis[7], Sarah L. Gaffen[4], Nicholas Jendzjowsky[2,3,8], Scott G. Filler[1,2,8], Marc Swidergall[1,2,8*]

Affiliations
[1]Division of Infectious Diseases, Harbor-UCLA Medical Center, Torrance, CA, USA
[2]The Lundquist Institute for Biomedical Innovation at Harbor-UCLA Medical Center, Torrance, CA, USA
[3]Division of Respiratory and Critical Care Medicine and Physiology, Harbor-UCLA Medical Center, Torrance, CA, USA
[4]University of Pittsburgh, Division of Rheumatology and Clinical Immunology, Pittsburgh, PA, USA
[5]Graduate Program in Pharmaceutical Sciences, College of Graduate Health Sciences, University of Tennessee Health Science Center, Memphis, TN, USA
[6]Department of Clinical Pharmacy and Translational Science, College of Pharmacy, University of Tennessee Health Science Center, Memphis, TN, USA
[7]Fungal Pathogenesis Section, Laboratory of Clinical Immunology and Microbiology (LCIM), National Institute of Allergy and Infectious Diseases (NIAID), Bethesda, MD, USA
[8]David Geffen School of Medicine at UCLA, Los Angeles, CA, USA

*Correspondence: Marc Swidergall, mswidergall@lundquist.org

[†] Current address: Sorbonne Université, INSERM, Centre de Recherche Saint-Antoine (CRSA), Paris, France

Supplementary Material

- Supplementary Figures (1 – 27)

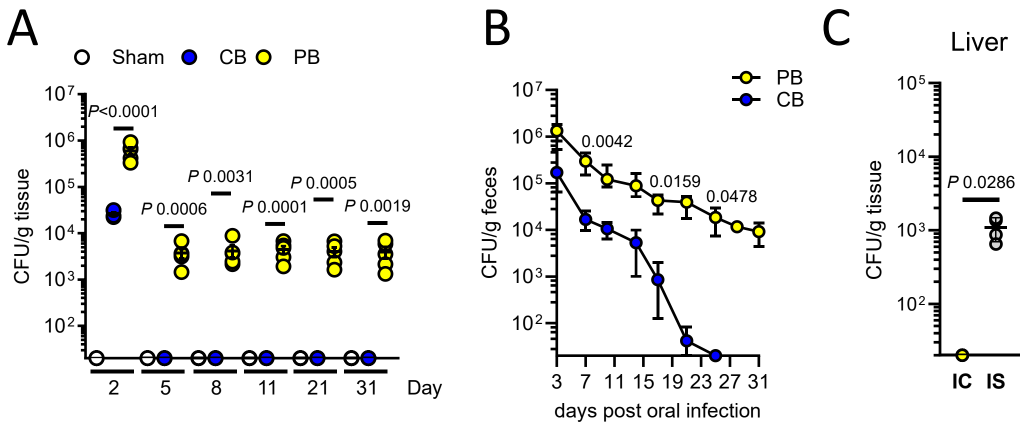

Supplementary Figure 1 The persistence-biased (PB) isolate CA101 persists in the oral cavity and GI tract following oral infection. **A** Oral fungal burden of wild-type mice infected with indicated strains. Two-tailed Mann-Whitney test. Results are median ± standard error of the mean (SEM) of four independent experiments (*N* = 5/group). **B** Fecal burden of wild-type mice after oral infection with indicated strains. The y-axis is set at the limit of detection (20 CFU/g tissue). Data are presented as mean values +/- range. N = 6 mice till day 14; 4 mice till day 21, 2 mice till day 31. Two-tailed Mann-Whitney test. **C** Liver fungal burden of immunocompetent (IC) and immunosuppressed (IS) wild-type mice infected with PB after 4 days of infection. Results are median ± standard error of the mean (SEM) of a single experiment (*N* = 4/group). CB, clearance-biased; PB, persistence-biased.

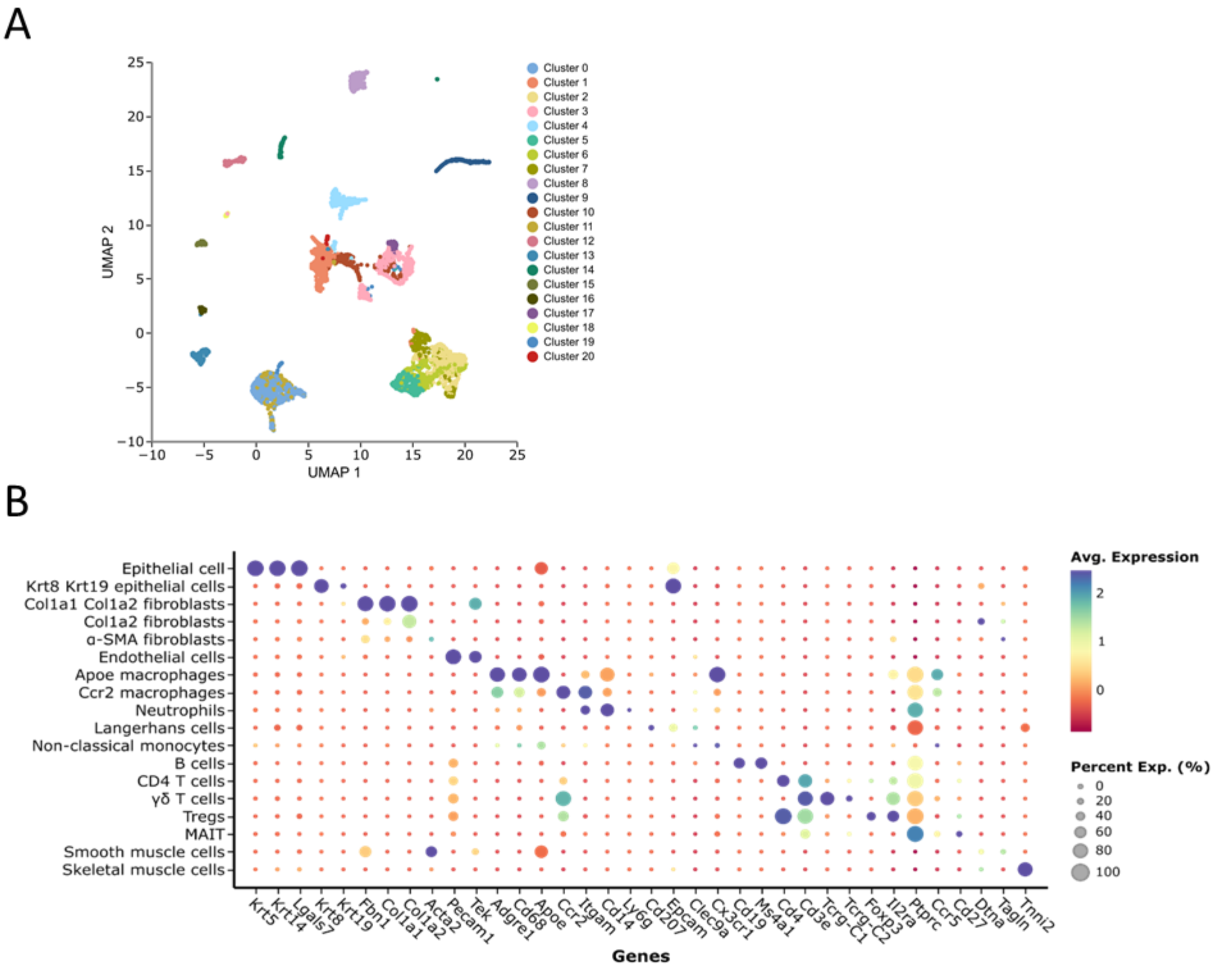

Supplementary Figure 2 Analysis of mucosal single cell RNA sequencing. A Louvain clustering of single cells isolated from the tongue of CB- and PB-infected mice after 11 days of infection. UMAP, Uniform Manifold Approximation and Projection for Dimension Reduction. **B** Dot plot of scaled cell-type specific marker gene expression of identified subpopulations.

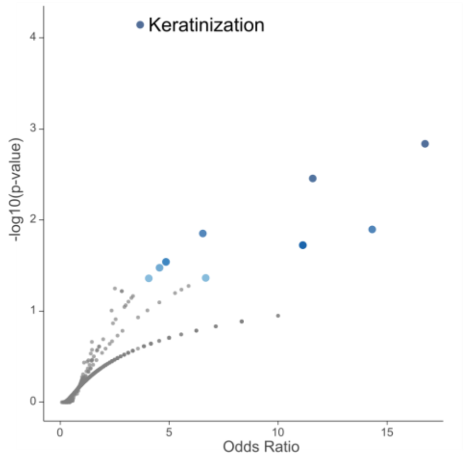

Supplementary Figure 3 Enriched pathways in epithelial cells from colonized mice in the scRNA-sequencing data set. Upregulated genes identified in the epithelial cell subset were analyzed for pathway enrichment. –log 10 p value is plotted against the odds ratio.

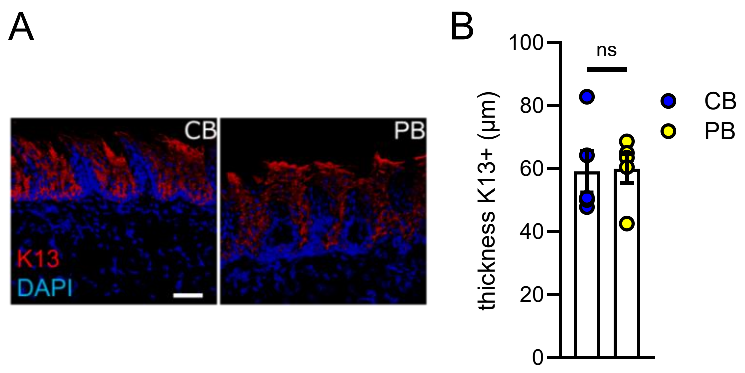

Supplementary Figure 4 K13 distribution remains the same during PB-colonization. A Representative immunofluorescence pictures of keratin 13 (K13) after 11 days of infection. Mice were infected with CB and PB. Scale bar 50µm. **B** K13 thickness 11 days post infection. *N* = 5. Two-tailed Mann-Whitney. Data are presented as mean values +/- SEM. For each animal (five mice per group), one section per tongue was randomly selected and stained.

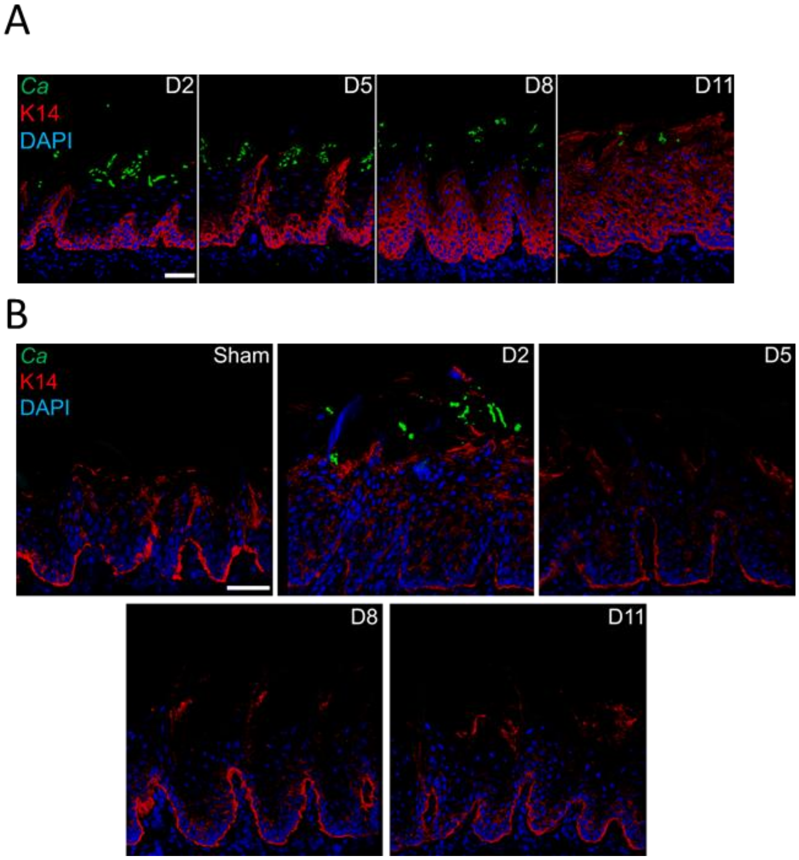

Supplementary Figure 5 Time course studies of K14 epithelial expansion. Representative immunofluorescence pictures of keratin 14 (K14) after indicated time points. Mice were infected with PB **A** or CB strain **B**. Scale bar 50µm.

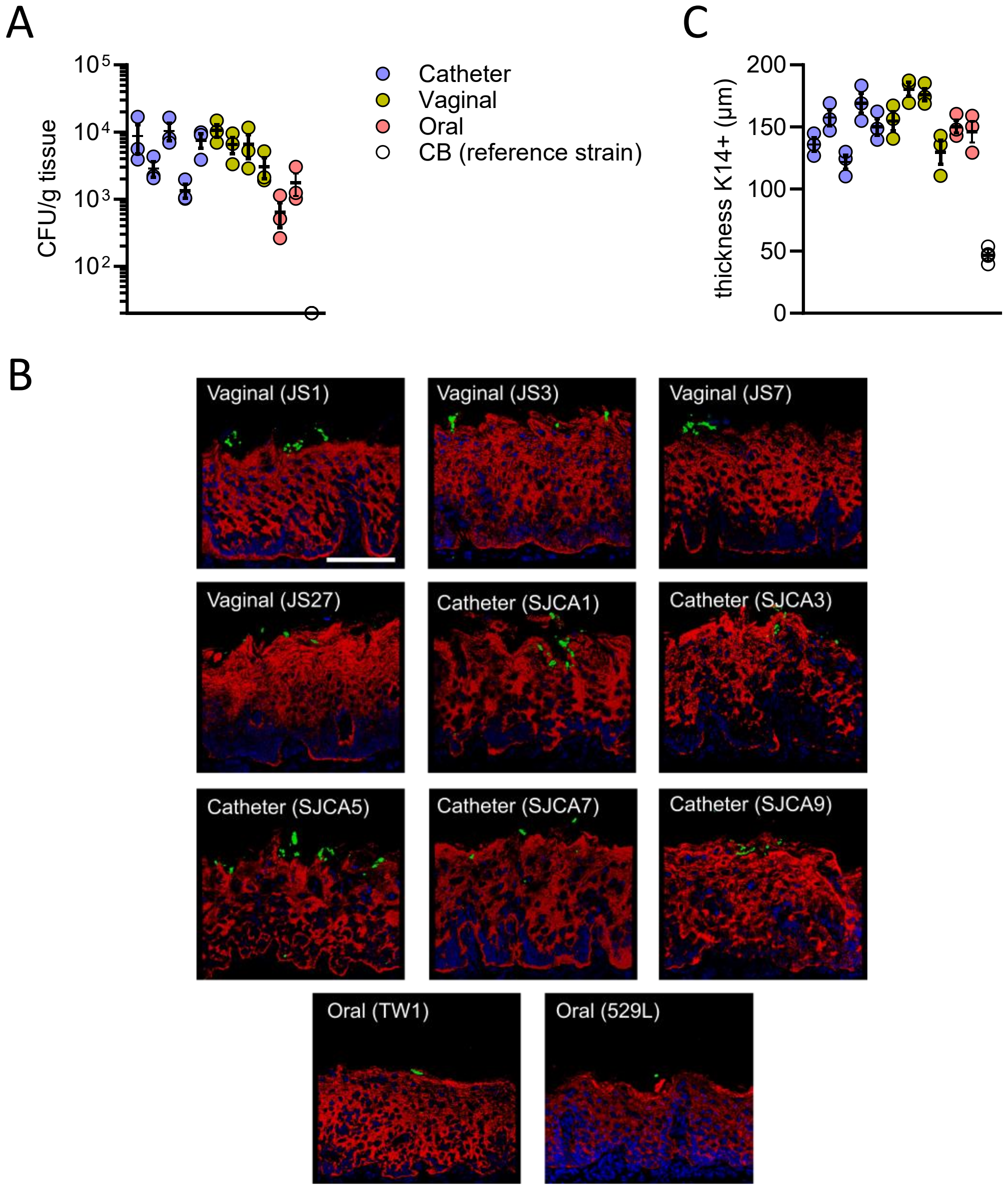

Supplementary Figure 6 ***C. albicans*** **clinical isolates show PB phenotype in the oral mucosa. A** Oral fungal burden of wild-type mice infected with indicated strains. Results are median ± standard error of the mean (SEM) of a single experiment (*N* = 3/group). **B** Representative immunofluorescence pictures of keratin 14 (K14) after 11 days of infection with indicated strains. Scale bar 100μm. **C** K14 thickness 11 days post infection with indicated strains. *N* = 3/group.

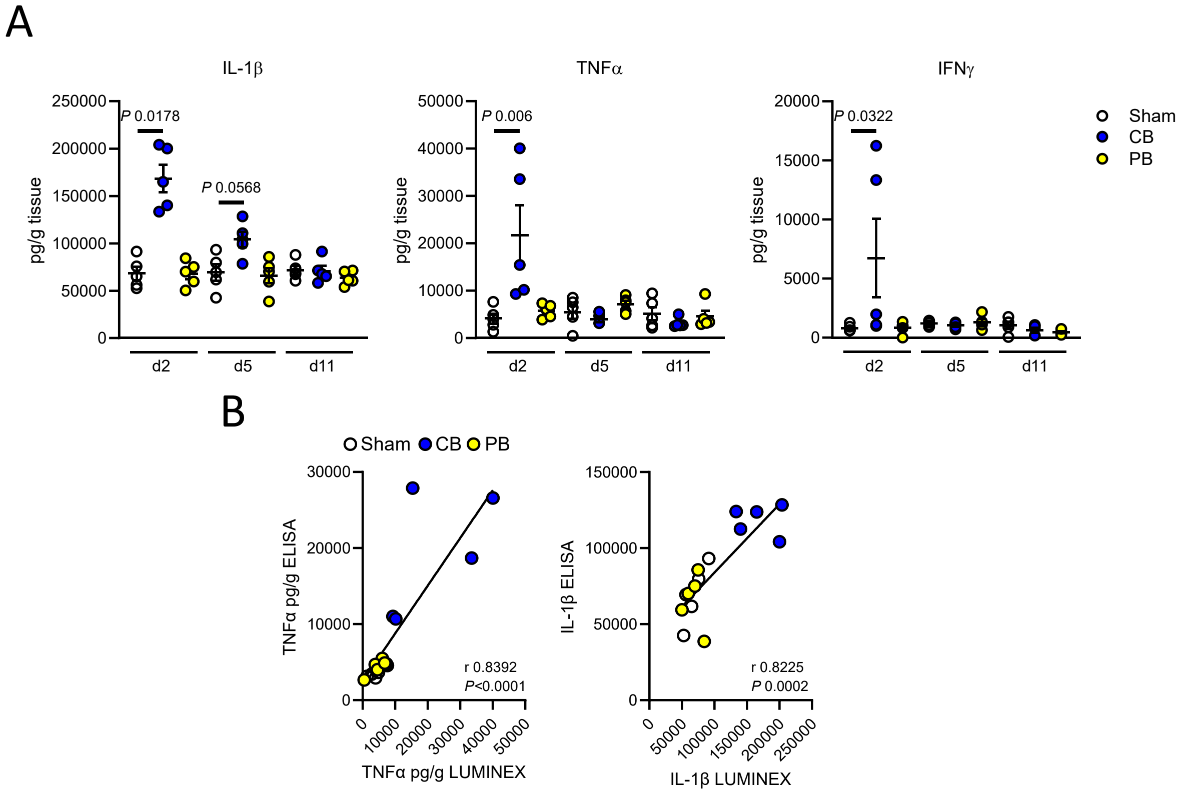

Supplementary Figure 7 Proinflammatory cytokines during OPC. A Levels of IL-1β, TNFα and IFNγ in CB-, PB-, and Sham-infected mice over time; *N* = 5. Ordinary one-way ANOVA. Data are presented as mean values +/- SEM. **B** Correlation of LUMINEX cytokine values and cytokines measured via ELISA at day 2 of oral infection. Correlation was determined by Pearson.

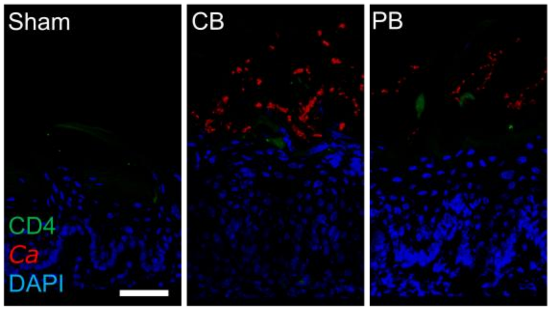

Supplementary Figure 8 Representative immunofluorescence pictures of CD4 expression after 2 days of infection. Mice were infected with CB and PB. Scale bar 50μm. Each experiment was repeated independently two times with similar results.

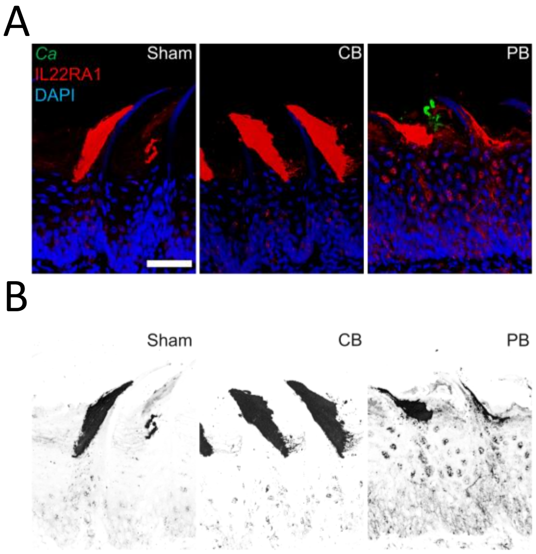

Supplementary Figure 9 IL-22RA1 distribution during fungal colonization in the oral cavity. **A** Representative immunofluorescence pictures of IL-22RA1 after 11 days of infection. Mice were infected with CB, PB, and Sham. Scale bar 50µm. **B** Red channel IL-22RA1 shown in black. Each experiment was repeated independently three times with similar results.

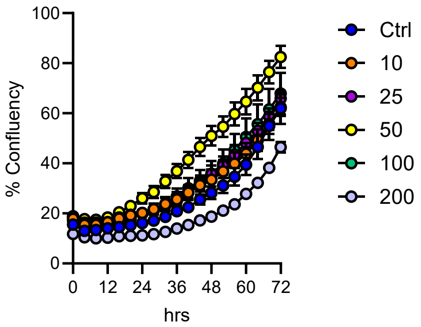

Supplementary Figure 10 Effect of different doses of IL-22 on oral epithelial cell proliferation. Growth curve of human oral epithelial cells in the presence of different amounts recombinant IL-22. *N*=8; independent cultures.

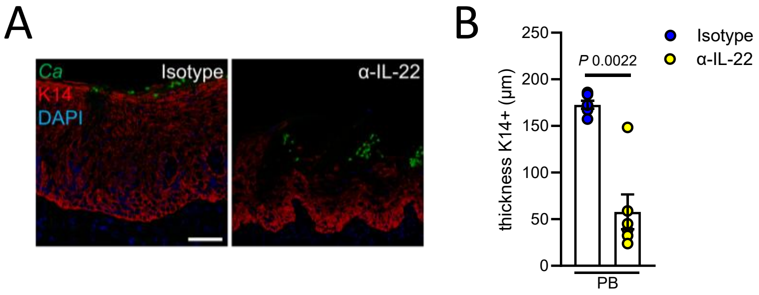

Supplementary Figure 11 Effect of IL-22 depletion on K14 expansion during oral fungal colonization A Representative immunofluorescence pictures of keratin 14 (K14) after 11 days of infection. Mice were infected with the PB strain and treated with isotype or α-IL-22 antibody starting day -1 every other day. Scale bar 50µm. Each experiment was repeated independently two times with similar results. **B** Quantification of K14 thickness in indicated mice on day 11. *N* = 6; combined data of two independent experiments. Two-tailed Mann-Whitney Test. Data are presented as mean values +/- SEM.

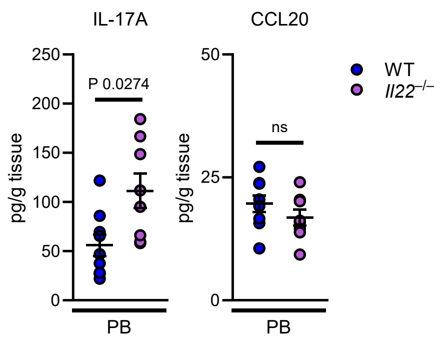

Supplementary Figure 12 Intact IL-17 signaling in *Il22*$^{-/-}$ mice during *Candida* colonization. Levels of IL-17A and CCL20 in tongue homogenates after 11 days of PB colonization. Combined data of two independent experiments. N=8-9. Two-tailed Mann-Whitney. Data are presented as mean values +/- SEM.

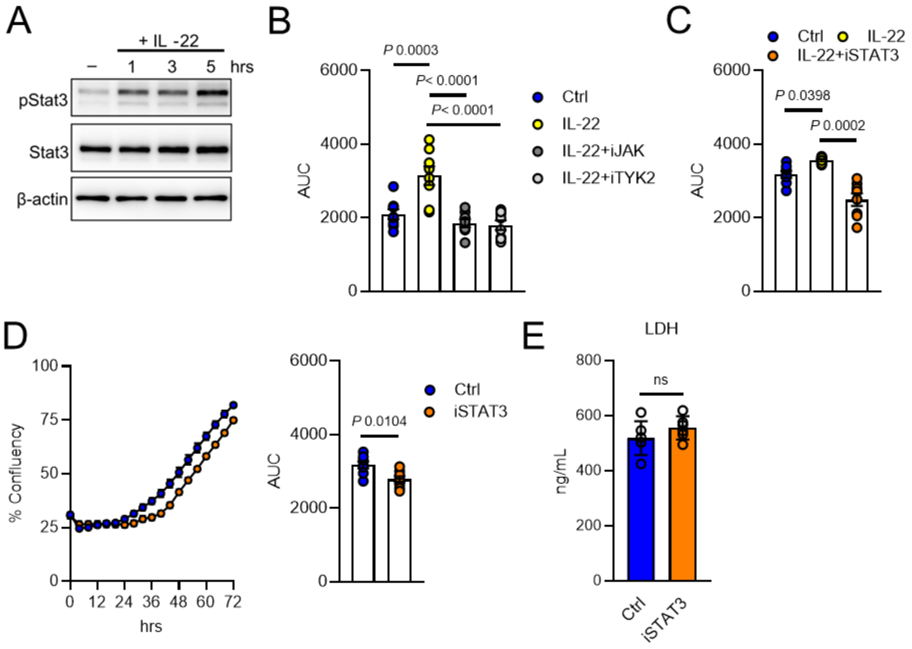

Supplementary Figure 13 JAK-STAT3 signaling is required for oral epithelial proliferation. A Representative immunoblot of STAT3 activation during IL-22 incubation with 50ng/ml. Each experiment was repeated independently three times with similar results.**B** Area under the curve (AUC) of oral epithelial cells in response to IL-22 treatment in the presence of JAK inhibitor (iJak) and TYK2 inhibitor (iTyk2). Growth was determined by confluence over time. *N* = 8; individual cultures. Ordinary one-way ANOVA. **C** Area under the curve (AUC) of oral epithelial cells in response to IL-22 treatment in the presence of STAT3 inhibitor (iSTAT3) and Tyk2 inhibitor (iTYK2). Growth was determined by confluence over time. *N* = 8; individual cultures. Ordinary one-way ANOVA. **D** Growth curve of human oral epithelial cells in the presence of STAT3 inhibitor. *N*=8; individual cultures. AUC of oral epithelial cells in response to iSTAT3 treatment. Growth was determined by confluence over time. *N* = 8; individual cultures. Two-tailed Mann-Whitney Test. **E** Lactate dehydrogenase (LDH) release of iSTAT3 treated oral epithelial cells after 8 hours. Two-tailed Mann–Whitney Test. *N* = 3 in duplicate.

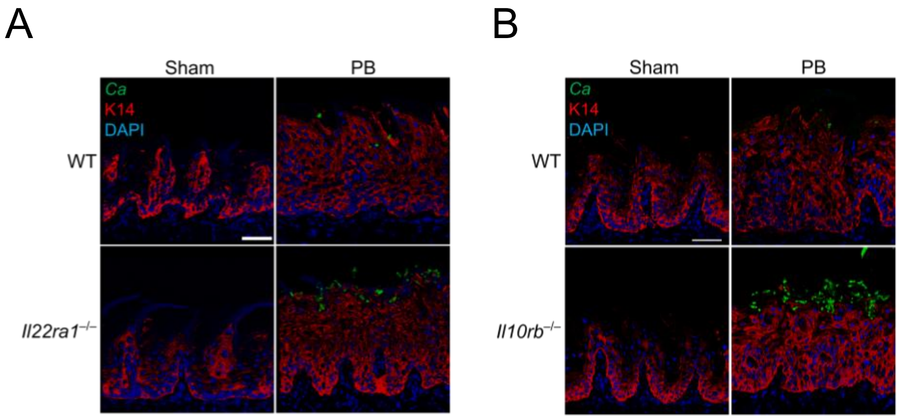

Supplementary Figure 14 K14 epithelial expansion during PB-colonization in *Il22ra1*$^{-/-}$ and *Il10rb*$^{-/-}$ mice. Representative immunofluorescence pictures of K14 after 11 days of Sham- or PB infection of *Il22ra1*$^{-/-}$ (**A**) and *Il10rb*$^{-/-}$ (**B**) mice. Scale bar 50µm. Each experiment was repeated independently two times with similar results.

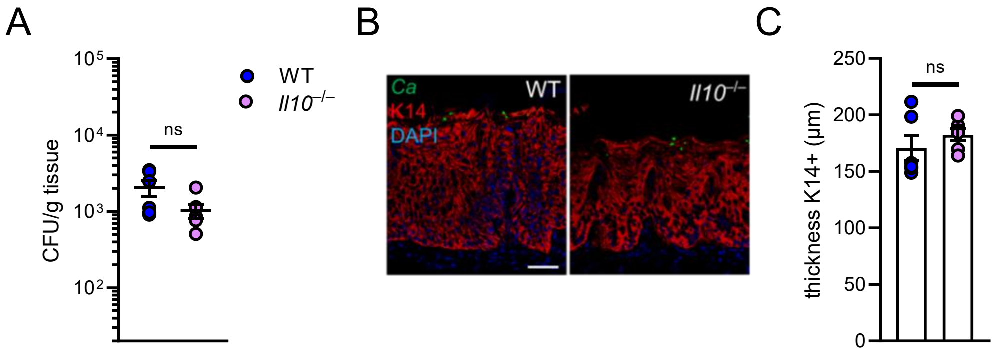

Supplementary Figure 15 IL-10 is dispensable to control oral fungal burden and K14 expansion during PB-colonization. A Oral fungal burden of WT and *Il10*$^{-/-}$ mice colonized with PB after 11 days. *N* = 6; combined data of two independent experiments. Two-tailed Mann–Whitney Test. Data are presented as mean values +/- SEM. **B** Representative immunofluorescence pictures of K14 after 11 days of infection of indicated mice. Mice were infected with PB. Scale bar 50 µm. Each experiment was repeated independently two times with similar results.**C** Quantification of K14 thickness in indicated mice at day 11. *N* = 6; combined data of two independent experiments. Two-tailed Mann–Whitney Test. Data are presented as mean values +/- SEM.

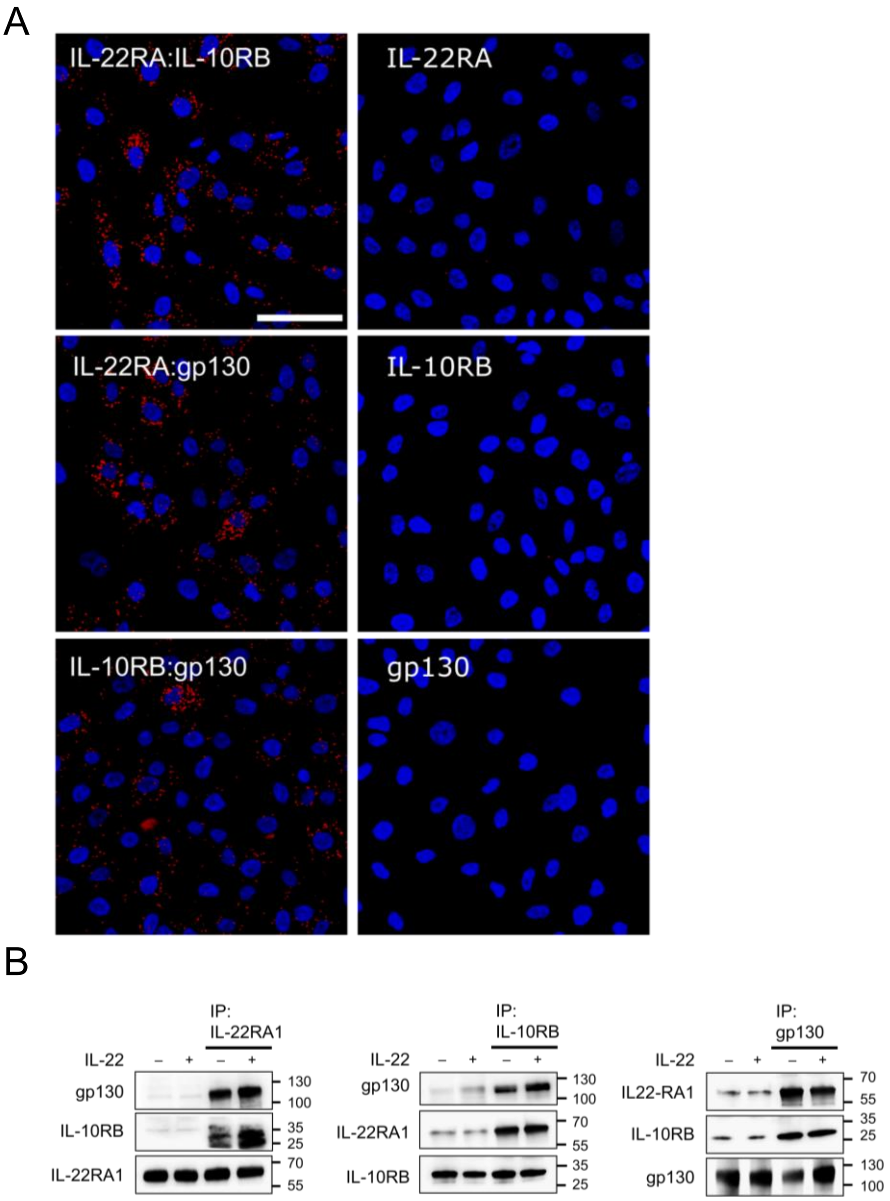

Supplementary Figure 16 gp130 forms receptor complexes with IL-10RB and IL-22RA1. A Representative images for proximity ligation assay (PLA) for indicated receptor complexes. Single antibody incubation was used as control Scale bar 50µm. Red dots indicate receptor complexes. **B** Representative immunoblot of immunoprecipitation of receptor complexes. OKF6-TERT2 cells were stimulated for 180 min with 50 ng/ml IL-22. After lysis, proteins (IL-22RA1, IL-10RB, and gp130) were immune-precipitated (IP) using specific antibodies. Lysates and pull-down samples (IP) were analyzed by immunoblotting. *N* = 3. Each experiment was repeated independently three times with similar results.

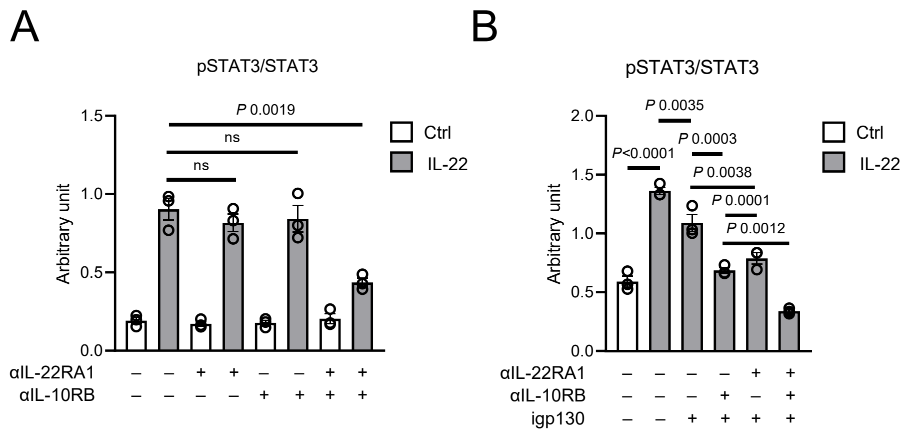

Supplementary Figure 17 Quantification of STAT3 activity during IL-22 incubation. A Densitometric analysis (arbitrary units) of STAT3 phosphorylation normalized to total STAT3 protein in the presence of IL-22RA1, IL-10RB, or combination of IL-22RA1/IL-10RB blocking antibodies. *N* = 3. Ordinary one-way ANOVA. Data are presented as mean values +/- SEM. **B** Densitometric analysis (arbitrary units) of STAT3 phosphorylation normalized to total STAT3 protein in the presence of IL-22RA1, IL-10RB, igp130 (gp130 inhibitor), or combination. *N* = 3. Ordinary one-way ANOVA. Data are presented as mean values +/- SEM.

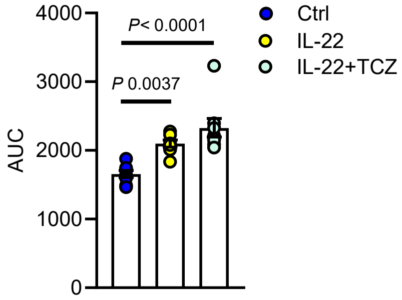

Supplementary Figure 18 Oral epithelial cell proliferation in the presence of IL-6 receptor blocker tocilizumab. Area under the curve (AUC) of oral epithelial cells in response to IL-22 treatment in the presence of tocilizumab. Growth was determined by confluence over time. N = 8; individual cultures. Ordinary one-way ANOVA. Data are presented as mean values +/- SEM. TCZ, tocilizumab.

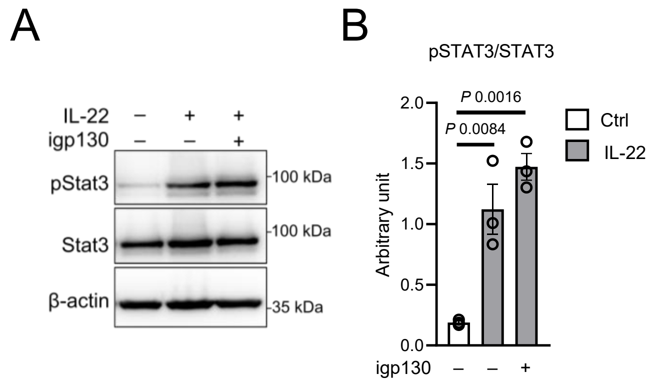

Supplementary Figure 19 STAT3 activation in intestinal epithelial cells is independent of gp130 signaling. A Representative immunoblot of STAT3 activation in intestinal epithelial cells during IL-22 incubation in the presence of gp130 inhibitor. Each experiment was repeated independently three times with similar results. **B** Densitometric analysis (arbitrary units) of STAT3 phosphorylation normalized to total STAT3 protein in the presence of igp130 (gp130 inhibitor) in intestinal epithelial cells. *N* = 3. Ordinary one-way ANOVA. Data are presented as mean values +/- SEM.

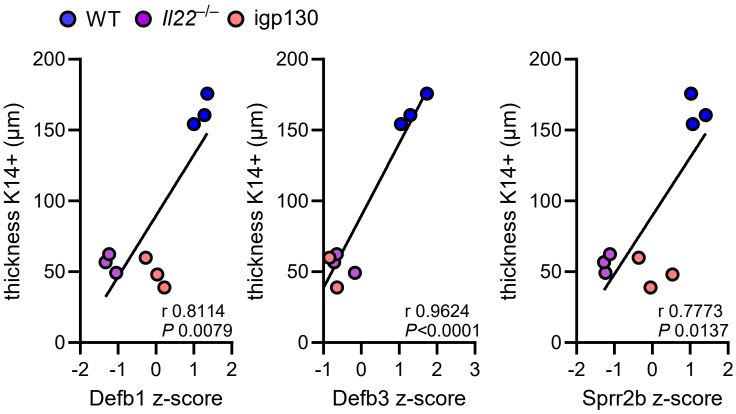

Supplementary Figure 20 Epithelial thickness correlates with antimicrobial peptide expression. Correlation of K14 thickness and antimicrobial peptide genes (Defb1, Defb3, Sprr2b). *N* = 3. Correlation was determined by Pearson.

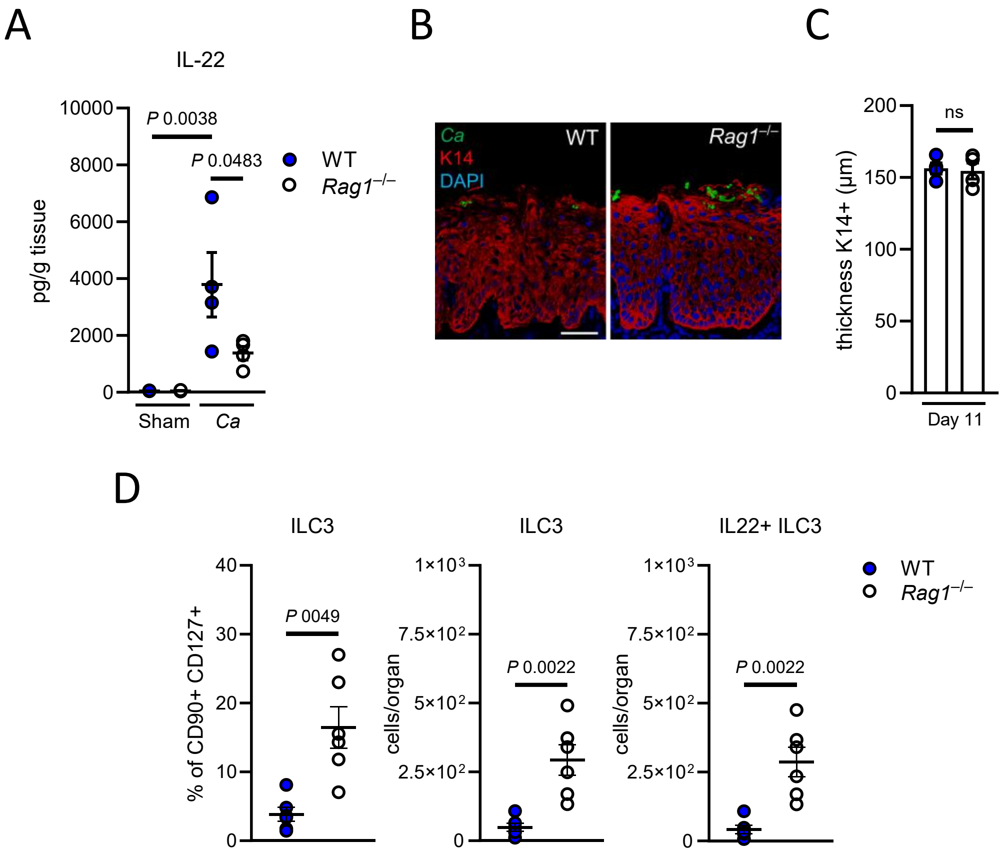

Supplementary Figure 21 *Rag1*$^{-/-}$ mice express IL-22 in the oral mucosa during PB colonization. A IL-22 levels in PB-, and Sham-infected mice; *N* = 4. Ordinary one-way ANOVA corrected for multiple comparison. Data are presented as mean values +/- SEM. **B** Representative pictures of K14 staining of Sham- and PB-infected mice after 11 days. Scale bar 50µm. **C** Quantification of K14 thickness in Sham- and PB-infected WT and *Rag1*$^{-/-}$ mice at day 11. *N* = 4. Data are presented as mean values +/- SEM. **D** Percentage (%, left), total (middle), and IL-22^{+} ILC3s (right) in PB-infected WT and *Rag1*$^{-/-}$ mice at day 11. *N* = 6. Two-tailed Mann–Whitney Test. Data are presented as mean values +/- SEM.

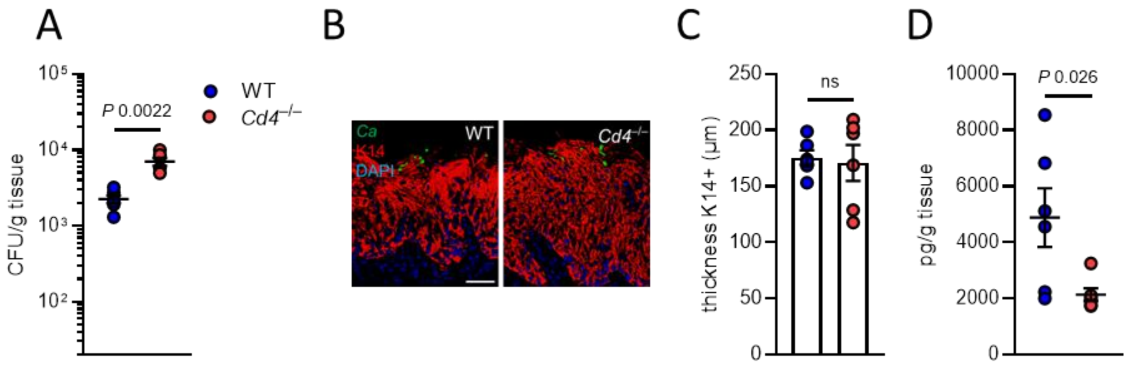

Supplementary Figure 22 *Cd4*$^{-/-}$ mice are more susceptible to PB infection and epithelial remodeling occurs in the absence of CD4 T cells. Oral fungal burden of indicated mice colonized with PB. *N* = 6; combined data of two independent experiments. Two-tailed Mann–Whitney Test. The y-axis is set at the limit of detection (20 CFU/g tissue). Data are presented as mean values +/- SEM. **B** Representative pictures of K14 staining of PB-infected mice after 11 and 31 days. Scale bar 50µm. Each experiment was repeated independently two times with similar results. **C** Quantification of K14 thickness in PB-infected mice at indicated time points. *N* = 6; combined data of two independent experiments. Two-tailed Mann–Whitney Test. Data are presented as mean values +/- SEM. **D** IL-22 levels in PB-infected mice; *N* = 6. Two-tailed Mann–Whitney Test. Data are presented as mean values +/- SEM.

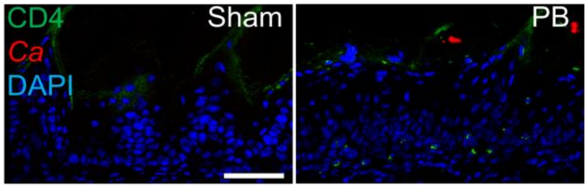

Supplementary Figure 23 CD4 T cell infiltration at the onset of neonatal colonization. Representative immunofluorescence pictures of CD4 cells after 12 days of PB colonization of 2-day-old neonates mice. Scale bar 50μm. Each experiment was repeated independently two times with similar results.

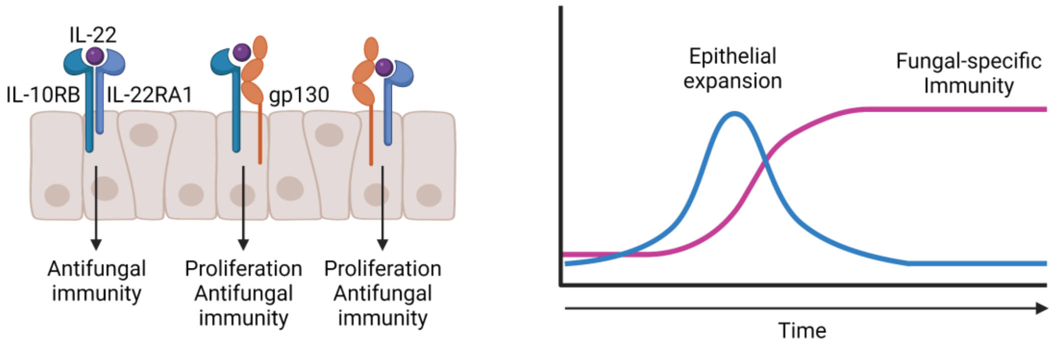

Supplementary Figure 24 Commensal fungi remodel the mucosal epithelial barrier. Left: IL-22 responsive receptor complexes promoting antifungal immunity and epithelial remodeling. Right: Fungal colonization generates a primary epithelial remodeling response followed by the generation of fungal-specific immunity and auxiliary epithelial remodeling. IL-22, interleukin-22; IL-10RB, interleukin 10 receptor subunit beta; Il-22RA1, interleukin 22 receptor subunit alpha 1; gp130, glycoprotein 130. Created in BioRender. Swidergall, M. (2026) https://BioRender.com/n7bcr96.

A

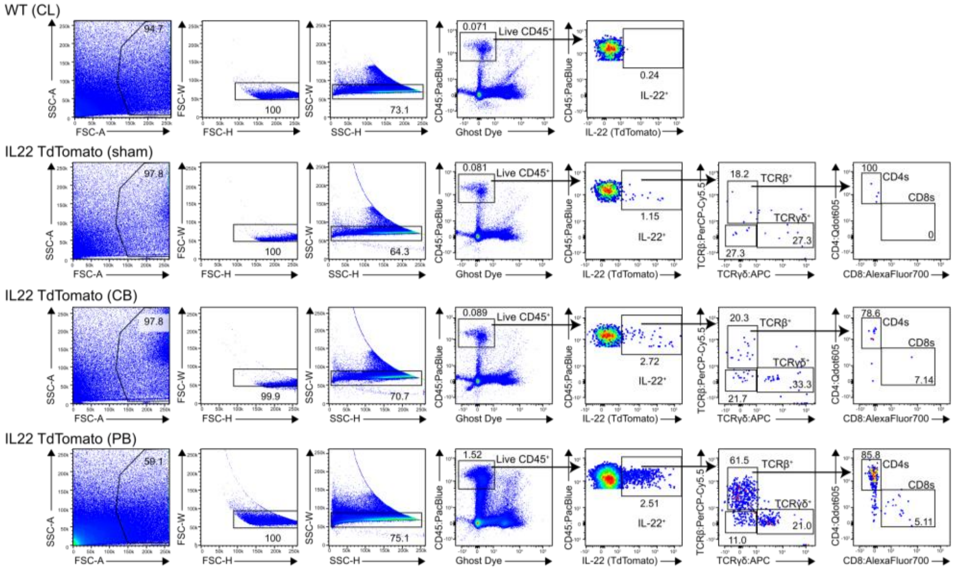

WT (CL)
IL22 TdTomato (sham)
IL22 TdTomato (CB)
IL22 TdTomato (PB)
SSC-A
FSC-A
FSC-W
FSC-H
SSC-W
SSC-H
CD45:PacBlue
Ghost Dye
Live CD45⁺
IL-22 (TdTomato)
IL-22⁺
TCRβ:PerCP-Cy5.5
TCRγδ:APC
TCRβ⁺
TCRγδ⁺
CD4:Qdot605
CD8:AlexaFluor700
CD4s
CD8s
94.7
100
73.1
0.071
0.24
97.8
100
64.3
0.081
1.15
18.2
27.3
27.3
100
0
97.8
99.9
70.7
0.089
2.72
20.3
21.7
33.3
78.6
7.14
59.1
100
75.1
1.52
2.51
61.5
11.0
21.0
85.8
5.11

B

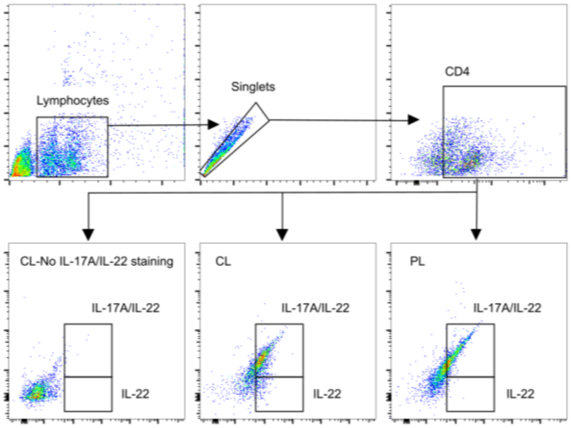

Lymphocytes
Singlets
CD4
CL-No IL-17A/IL-22 staining
CL
PL
IL-17A/IL-22
IL-22

C

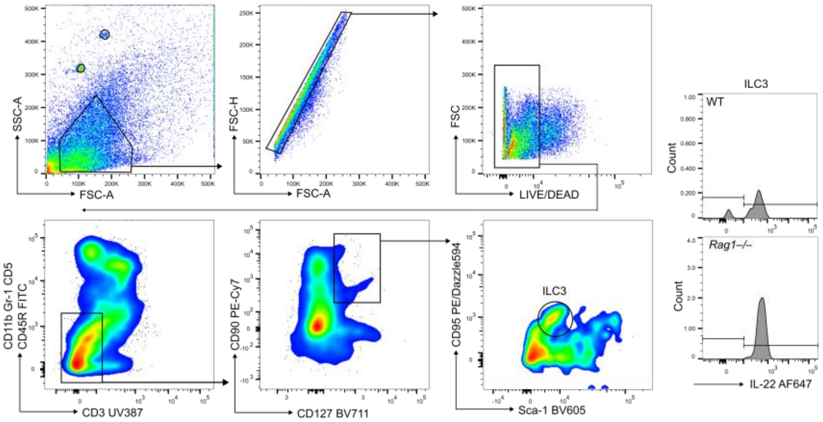

SSC-A
FSC-A
FSC-H
FSC
LIVE/DEAD
ILC3
WT
Rag1–/–
Count
IL-22 AF647
CD11b Gr-1 CD5
CD45R FITC
CD3 UV387
CD90 PE-Cy7
CD127 BV711
CD95 PE/Dazzle594
Sca-1 BV605

Supplementary Figure 25 Gating strategy of immune cells in the oral mucosa. A Gating strategy in *Il22TdTomato* mice. Cells were identified as singlets live IL-22+ and further gated on TCRβ, TCTγδ, CD4, and CD8. **B** Th17 cells were identified as singlets CD4+ IL-17A+ IL22+ and Th22 cells were identified as singlets CD4+ IL-17A- IL22+. **C** ILC3 cells were identified as singlets live Gr-1- CD5- CD11b- CD45R- CD3- CD90+ CD127+ Sca-1+ CD95+.

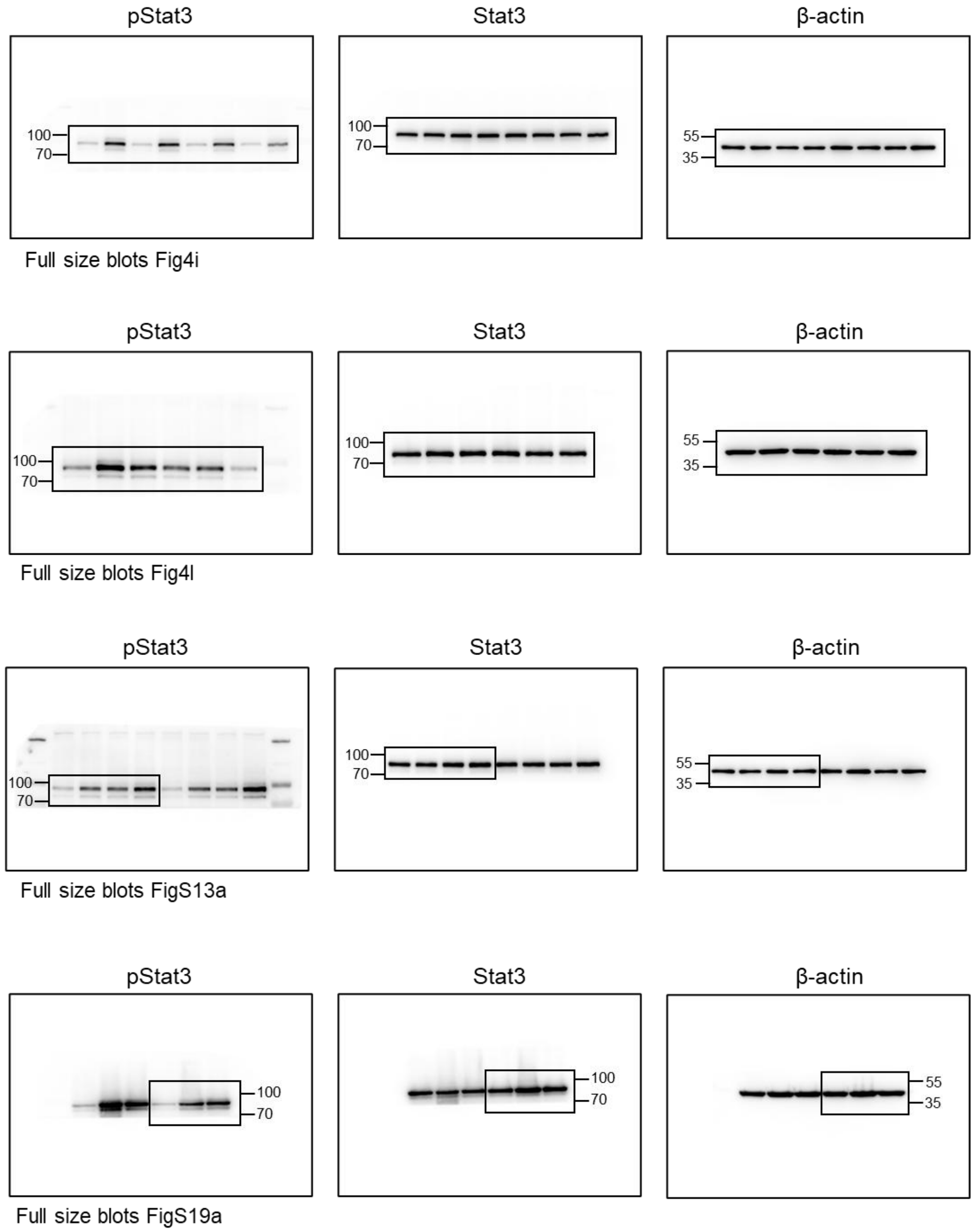

Supplementary Figure 26 Uncropped immunoblots. Uncropped immunoblots of indicated figures.

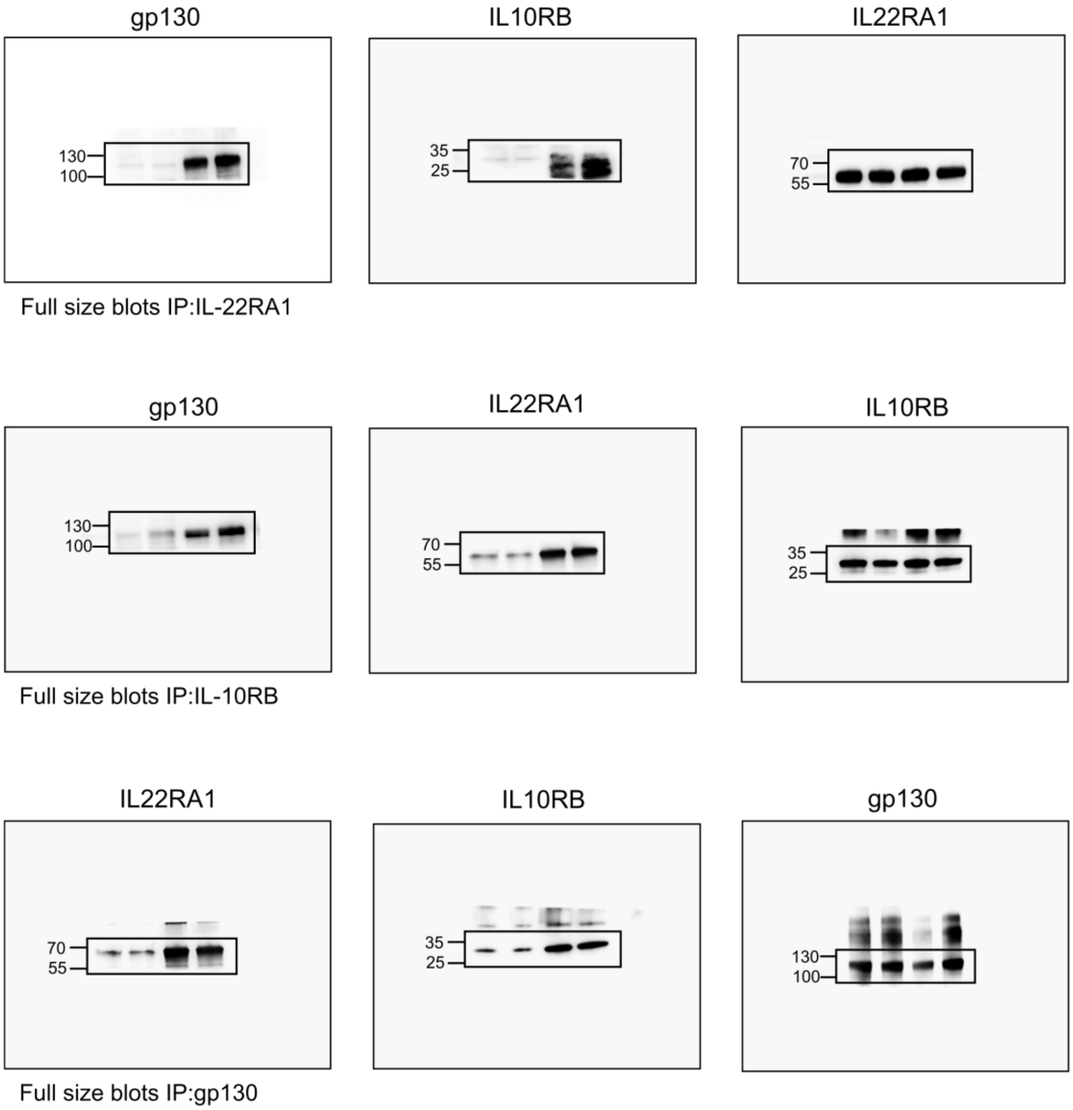

Supplementary Figure 27 Uncropped immunoblots of immunoprecipitation. Uncropped immunoblots of indicated IPs.
