# Supplementary material for: Non-canonical IL‑22 receptor signaling remodels the oral mucosal barrier during Candida albicans immunosurveillance
Source: Nat Commun. 2026 Apr 3;17:4823. doi: 10.1038/s41467-026-71459-z (PMC13223278; doi:10.1038/s41467-026-71459-z)
Supplement: Supplementary file 2 — Reporting Summary [file 41467_2026_71459_MOESM2_ESM.pdf]

nature portfolio

Corresponding author(s): Marc Swidergall

Last updated by author(s): Feb 2, 2026

Reporting Summary

Nature Portfolio wishes to improve the reproducibility of the work that we publish. This form provides structure for consistency and transparency in reporting. For further information on Nature Portfolio policies, see our Editorial Policies and the Editorial Policy Checklist.

Statistics

For all statistical analyses, confirm that the following items are present in the figure legend, table legend, main text, or Methods section.

n/a	Confirmed	
☐	☒	The exact sample size (n) for each experimental group/condition, given as a discrete number and unit of measurement
☐	☒	A statement on whether measurements were taken from distinct samples or whether the same sample was measured repeatedly
☐	☒	The statistical test(s) used AND whether they are one- or two-sided<br>*Only common tests should be described solely by name; describe more complex techniques in the Methods section.*
☒	☐	A description of all covariates tested
☐	☒	A description of any assumptions or corrections, such as tests of normality and adjustment for multiple comparisons
☐	☒	A full description of the statistical parameters including central tendency (e.g. means) or other basic estimates (e.g. regression coefficient) AND variation (e.g. standard deviation) or associated estimates of uncertainty (e.g. confidence intervals)
☐	☒	For null hypothesis testing, the test statistic (e.g. F, t, r) with confidence intervals, effect sizes, degrees of freedom and P value noted<br>*Give P values as exact values whenever suitable.*
☒	☐	For Bayesian analysis, information on the choice of priors and Markov chain Monte Carlo settings
☒	☐	For hierarchical and complex designs, identification of the appropriate level for tests and full reporting of outcomes
☐	☒	Estimates of effect sizes (e.g. Cohen's d, Pearson's r), indicating how they were calculated

Our web collection on statistics for biologists contains articles on many of the points above.

Software and code

Policy information about availability of computer code

Data collection	N/A
Data analysis	N/A

For manuscripts utilizing custom algorithms or software that are central to the research but not yet described in published literature, software must be made available to editors and reviewers. We strongly encourage code deposition in a community repository (e.g. GitHub). See the Nature Portfolio guidelines for submitting code & software for further information.

Data

Policy information about availability of data

All manuscripts must include a data availability statement. This statement should provide the following information, where applicable:

- Accession codes, unique identifiers, or web links for publicly available datasets
- A description of any restrictions on data availability
- For clinical datasets or third party data, please ensure that the statement adheres to our policy

The high-throughput sequencing data from this study have been submitted to the NCBI Sequence Read Archive (SRA) under accession number PRJNA1176644

Research involving human participants, their data, or biological material

Policy information about studies with human participants or human data. See also policy information about sex, gender (identity/presentation), and sexual orientation and race, ethnicity and racism.

Reporting on sex and gender	N/A
Reporting on race, ethnicity, or other socially relevant groupings	N/A
Population characteristics	N/A
Recruitment	N/A
Ethics oversight	N/A

Note that full information on the approval of the study protocol must also be provided in the manuscript.

Field-specific reporting

Please select the one below that is the best fit for your research. If you are not sure, read the appropriate sections before making your selection.

[x] Life sciences [ ] Behavioural & social sciences [ ] Ecological, evolutionary & environmental sciences

For a reference copy of the document with all sections, see nature.com/documents/nr-reporting-summary-flat.pdf

Life sciences study design

All studies must disclose on these points even when the disclosure is negative.

Sample size	For in vivo experiments (fungal burden, immune cell recruitment, cytokines) we will use 6 mice (Knockouts or treatment) and 6 controls. In previous studies the response within each subject group was normally distributed with standard deviation 0.25. If the true difference in the experimental and control means is 0.5 (e.g., 50% increase or decrease), we will be able to reject the null hypothesis that the population means of the experimental and control groups are equal with probability (power) >0.85. The Type I error probability associated with this test of this null hypothesis is 0.05. All experiments used power analysis to determine the appropriate number of mice.<br>At least three biological replicates were performed for all in vitro experiments unless otherwise indicated. Data were compared by Mann-Whitney or ANOVA corrected for multiple comparisons using GraphPad Prism (v. 10) software. P values < 0.05 were considered statistically significant.
Data exclusions	No data were excluded
Replication	All experimental findings were reproduced (in vivo: at least two times; in vitro at least three times)
Randomization	gp130 inhibitor, antibody-depletion and CD4 T cell transfer: The mice were randomized to the two different treatment groups (drug or antibody vs. vehicle or primed T cells vs. naive).
Blinding	Researchers were not blinded to the experimental groups because the endpoints (fungal burden, cytokine levels, etc.) were objective measures of disease severity. However, the outcomes were quantitative and not subjective.

Reporting for specific materials, systems and methods

We require information from authors about some types of materials, experimental systems and methods used in many studies. Here, indicate whether each material, system or method listed is relevant to your study. If you are not sure if a list item applies to your research, read the appropriate section before selecting a response.

Materials & experimental systems

n/a	Involved in the study	
[ ]	[x]	Antibodies
[ ]	[x]	Eukaryotic cell lines
[x]	[ ]	Palaeontology and archaeology
[ ]	[x]	Animals and other organisms
[x]	[ ]	Clinical data
[x]	[ ]	Dual use research of concern
[x]	[ ]	Plants

Methods

n/a	Involved in the study	
[x]	[ ]	ChIP-seq
[ ]	[x]	Flow cytometry
[x]	[ ]	MRI-based neuroimaging

Antibodies

Antibodies used

pSTAT3 (Tyr705, D3A7, #9145, Cell Signaling)
STAT3 (D3Z2G, #12640, Cell Signaling)
!-actin (8H10D10, #3700, Cell Signaling)
K14 (#Ab181595, Abcam)
K13 (#BSM-52053R, Bioss)
Ki67 (#9126S, Cell Signaling)
CD4 (#100405, Biolegend)
IL17A (#506915; Biolegend)
IL22 (#12-7221-80; eBiosciences)
Ly-6G/Ly-6C (FITC, RB6-8C5, #108405, Biolegend)
CD5 (FITC, 53-7.3, #100605, Biolegend)
CD11b (FITC, M1/70, #101205, Biolegend)
CD45R (FITC, RA3-6B2, #103205, Biolegend)
CD3 (Spark UV 387, 17A2, #100283, Biolegend)
CD90 (PE-Cy7, W20280E, #166409, Biolegend)
Ly-6A/Ly-6E (PerCP, D7, #108121, Biolegend)
CD127 (Brilliant Violet 711, SB/199, #121125, Biolegend)
CD95 (PE-Dazzle 594, SA367H8, #152625, Biolegend)
IL-22 (Alexa Fluor 647, Poly5164, #516406, Biolegend)

Validation

All antibodies were purchased from commercial sources.

Eukaryotic cell lines

Policy information about cell lines and Sex and Gender in Research

Cell line source(s)

OKF2-TERT2 human oral epithelial cells

Authentication

Via RNAseq see also Conti, H. R. et al. IL-17 Receptor Signaling in Oral Epithelial Cells Is Critical for Protection against Oropharyngeal Candidiasis. Cell Host Microbe 20, 606-617 (2016)

Mycoplasma contamination

Tested and found to be uncontaminated.

Commonly misidentified lines
(See ICLAC register)

N/A

Animals and other research organisms

Policy information about studies involving animals; ARRIVE guidelines recommended for reporting animal research, and Sex and Gender in Research

Laboratory animals

-C57BL/6 control mice (wild type) purchased from The Jackson laboratory
-Il22ra1E2a-cre provided by Jay Kolls
-IL-22TdTomato provided by Scott K. Durum
-Il10rb–/– (B6.129S2-Il10rbtm1Agt/J) purchased from The Jackson laboratory
-Il22iCre/iCre (C57BL/6-Il22tm1.1(icre)Stck/J purchased from The Jackson laboratory
-Il10–/– (B6.129P2-Il10tm1Cgn/J) purchased from The Jackson laboratory
-Rag1–/– (B6.129S7-Rag1tm1Mom/J purchased from The Jackson laboratory

Wild animals

N/A

Reporting on sex

both sexes were used in experiments

Field-collected samples

N/A

Ethics oversight

All animal work was approved by the Institutional Animal Care and Use Committee (IACUC) of the Lundquist Institute at Harbor-UCLA Medical Center and University of Pittsburgh. #32371-01 #IS00017969

Note that full information on the approval of the study protocol must also be provided in the manuscript.

Plants

Seed stocks: N/A

Novel plant genotypes: N/A

Authentication: N/A

Flow Cytometry

Plots

Confirm that:

☒ The axis labels state the marker and fluorochrome used (e.g. CD4-FITC).

☒ The axis scales are clearly visible. Include numbers along axes only for bottom left plot of group (a 'group' is an analysis of identical markers).

☒ All plots are contour plots with outliers or pseudocolor plots.

☒ A numerical value for number of cells or percentage (with statistics) is provided.

Methodology

Sample preparation: Mice were orally infected with C. albicans as described above. After different time points, the animals were administered a sublethal anesthetic mix intraperitoneally. The thorax was opened, and a part of the rib cage removed to gain access to the heart. The vena cava was transected and the blood was flushed from the vasculature by slowly injecting 10 ml PBS into the right ventricle. The tongue was harvested and cut into small pieces in 100 µl of ice-cold PBS. 1 ml digestion mix (4.8 mg/ml Collagenase IV; Worthington Biochem, and 200 µg/ml DNase I; Roche Diagnostics, in 1x PBS) was added after which the tissue was incubated at 37°C for 30 min. The resulting tissue suspension was then passed through a 100 µm cell strainer. Cell suspensions were separated by Percoll gradient centrifugation as described before 15,36. To determine IL-17A and IL-22, cell suspensions were stimulated with Pharmingen™ Leukocyte Activation Cocktail (BD Biosciences) for 5 hours. For Th17 and Th22 cells, cells were washed and stained with CD4 antibody (BioLegend), for ILC3 cells, cells were washed and stained with FITC conjugated Gr-1, CD5, CD11b, CD45R (to eliminate unwanted cell populations), and CD3, CD90, Sca-1, CD127, CD95 (BioLegend) antibodies. For intracellular staining, cells were fixed with Cytofix/Cytoperm (BD Biosciences) and stained for 1 hour with IL-17A (BioLegend) and IL-22 (BioLegend) antibodies. T

Instrument: BD FACSymphony™ A5 Cell Analyzer

Software: FACS Diva (BD Biosciences) and FlowJo software (Treestar)

Cell population abundance: After washing, negative CD4 T cell selection was performed using MojoSort™ Mouse CD4 T Cell Isolation Kit (#480006; BioLegend). Post-enrichment purity of CD4 T cells was >97.1%.

Gating strategy: he stained cells were analyzed on FACSymphony A5 system (BD Biosciences), and the data were analyzed using FACS Diva and FlowJo software. Th17 cells were identified as singlets CD4+ IL-17A+ IL22+, Th22 cells were identified as singlets CD4+ IL-17A- IL22+ and ILC3 cells were identified as singlets Gr-1- CD5- CD11b- CD45R- CD3- CD90+ CD127+ Sca-1+ CD95+

☒ Tick this box to confirm that a figure exemplifying the gating strategy is provided in the Supplementary Information.
